# Association of urinary sex hormones with mood and behavior changes in a community adolescent cohort

**DOI:** 10.1371/journal.pone.0293040

**Published:** 2023-10-16

**Authors:** Philip Hazell, Ben W. R. Balzer, Frances Garden, David J. Handelsman, Karen Paxton, Catherine Hawke, Rebecca Ivers, S. Rachel Skinner, Georgina Luscombe, Katharine S. Steinbeck

**Affiliations:** 1 Faculty of Medicine and Health, The University of Sydney School of Medicine, Camperdown, NSW, Australia; 2 The University of New South Wales Faculty of Medicine, School of Women’s and Children’s Health, Randwick, NSW, Australia; 3 Sydney Children’s Hospital, Randwick, NSW, Australia; 4 Ingham Institute for Medical Research, The University of New South Wales, Liverpool, NSW, Australia; 5 The University of New South Wales Faculty of Medicine, School of Public Health and Community Medicine, Kensington, NSW, Australia; 6 Specialty of Child and Adolescent Health, The Children’s Hospital at Westmead Clinical School, Sydney, NSW, Australia; University of Rijeka Faculty of Medicine: Sveuciliste u Rijeci Medicinski fakultet, CROATIA

## Abstract

**Objective:**

To examine the contribution of variation in sex hormone excretion to mood and behavioral changes in adolescent females and males.

**Design:**

Prospective, longitudinal observational cohort study.

**Methods:**

Participants were 342 volunteers aged 10–12 years living in rural Australia. Urinary estradiol and testosterone levels measured by liquid chromatography-mass spectrometry were obtained at three-month intervals for three years. Integrated measures (area-under-curve) of urinary steroid excretion summarised as absolute and variability during each 12-month period of the study. Psychosocial data were gathered annually with the primary outcome of depressive symptomatology. Secondary outcomes were the other subscales of the Youth Self-Report, impulsive-aggression, sleep habits, and self-harm.

**Results:**

277 (158 male) participants contributed data over the full duration of the study and could be included in the analyses. In females, analyses of *absolute urine hormone levels* found no relationship between estradiol and any outcome, but higher testosterone was significantly associated with depression and poorer sleep. Greater *variability* of both urine estradiol and testosterone was associated with lower total psychopathology, anxious/depressed and social problems scores. Greater *variability* in urine estradiol was associated with lower attention problems and impulsive aggression in females. In males, higher testosterone and estradiol levels were associated with rule-breaking, and poorer sleep, and no associations were found for gonadal hormone variability for males.

**Conclusions:**

Longitudinal measurement of both iso-sexual and contra-sexual gonadal hormones contributes to a more nuanced view of the impact of sex steroids on mood and behavior in adolescents. These findings may enlighten the understanding of the impact of sex steroids during normal male and female puberty with implications for hormone replacement therapies as well as management of common mood and behavioral problems.

## Introduction

The impact of the major puberty hormones on adolescent mood and behavior based on empirical evidence is not well understood and remains controversial. Older studies have relied on cross-sectional data and less accurate steroid hormone immunoassays [1,2]. More recent longitudinal cohort studies focus more on the impact of early ontogeny or metabolic effects of puberty and usually lack the intensity of biological sampling required to decompose the contribution of normal puberty hormone changes on mood and behavior [3–7].

While it is certainly plausible that the dramatic changes in gonadal sex hormones during puberty may mediate dynamic changes in mood and behavior across adolescence, the relationship is likely to be complex [8,9]. The motivation of this study was to evaluate the association of pubertal hormonal changes with mood and behavior patterns. Despite a lack of confirmatory evidence, popular science writers, the lay community and some clinicians continue to express the stereotypical view that normal changes in testosterone during puberty produce aggression and out-of-control behaviour in males, and that estradiol produces depression and low mood in females–conceptualised as the ‘storm and stress’ hypothesis [10–13].

Our research aimed to determine the attributable impact of puberty hormone changes on adolescent mood and behavior in both sexes, based on intensively sampled sex hormone excretion during puberty with measures of mood and behavior in a community-based sample of adolescent males and females followed for three years. Our original hypothesis was the rate of change (speed/tempo) and/or the degree of change in gonadal hormones during puberty may explain the mood and behaviour changes observed during adolescence among individuals progressing through normal puberty [14].

## Materials and methods

The Adolescent Rural Cohort study of Hormones, health, Education, environments, and Relationships (ARCHER) was a five-year, prospective, multi-disciplinary longitudinal study with rolling recruitment. Its focus was on health and wellbeing in rural adolescents, centred on the regional cities of Orange and Dubbo in central western New South Wales, Australia. The detailed protocol is published [14]. Briefly, young adolescents aged between 10- and 12-years attending school in the region of interest and their parents were recruited via advertisement. This was not a study primarily about the timing of puberty, rather the age range selected was to ensure that most participants reached mid puberty where there is a marked rise in sex hormone production in both sexes. Participants were recruited and data collected between 2011 and 2016.

During this period individual participants provided, for three years, three-monthly *first morning fasted* urine specimens collected at home, yielding 13 data collection points in total. Post-menarcheal females provided samples in the mid-follicular phase (menstrual cycle days 7–10). Participants attended annual research office visits for anthropometry assessment, blood collection (fasting) and detailed survey completion. The approach afforded the rare opportunity to examine the cumulative influence of hormone change and variability on mood and behavior over time during puberty. However, as a community rather than clinical sample, ethical approval was denied to undertake pubertal staging by direct observation. As a result, we employed self-report against computerised Tanner line drawings as part of the annual survey. We have reported on relationships between Tanner staging and hormone results in this study which show longitudinal changes in self-rated Tanner stage were positively associated with changes in serum testosterone and estradiol in males and females, respectively (2), as well as wide variation in sex hormone excretion for any single Tanner stage.

### Hormone analyses

There was high adherence to providing urine samples so that only urine data are reported whereas adherence to blood sampling reduced significantly over time during the study. Congruence between urine and serum samples has been previously reported [15]. Hormone analysis was conducted on biological samples using a liquid chromatography-tandem mass spectrometry (LC-MS/MS) method using methods as reported [9,15]. Within-day and between-day reproducibility at three levels of quality control for each analyte ranged from 2.6–9.7%, respectively. LC-MS/MS is the gold standard for steroid hormone analysis, with higher sensitivity and specificity than steroid immunoassays [16] and is capable of detecting the low levels of circulating sex steroids typical of early puberty [15,17].

The limit of detection and quantitation for testosterone was 5 pg/mL and 25 pg/mL and for estradiol 25 pg/mL and 50 pg/ml, respectively (9). In a preliminary analysis in our cohort, 14 of 484 (3%) of urine sample were undetectable for estradiol and nine of 484 (2%) of urine testosterone measures were undetectable (10). Urine hormones were corrected for specific gravity (measured by an accredited pathology service on the day of initial urine collection) although first morning urine specimens appear unaffected by hydration status [18].

### Mood, behavior and sleep measures

At recruitment and at each annual clinic visit, adolescents, and a parent/carer each completed a survey encompassing a wide range of outcomes pertinent to the broader aims of ARCHER. Relevant to the present work, adolescents completed the Youth Self-Report (YSR), a 112-item validated self-report instrument which includes social competence items and eight sub-scales measuring internalizing and externalizing behaviors [19]. An impulsive-aggression measure was derived from YSR items 16, 20, 21, 37, 41, 57, 87, 95, 97, and 104, as per the method reported by Van Meter et al. [20] Adolescents completed the Short Moods and Feelings Questionnaire (SMFQ), a uni-dimensional measure of symptom severity of childhood depression [6]. Adolescents were also asked to self-report estimated sleep duration on weekdays and at weekends, as well as report on self-harm thoughts and behaviors. For this analysis the SMFQ and YSR anxious/depressed and withdrawn/depressed subscales were the primary outcome measures. Collection of mood, behavior and sleep measures was timed to occur within seven days of a urine sample.

### Statistical analysis

We estimated that 160 boys and 160 girls at Tanner Stage 3 would be required to detect a one standard deviation difference in testosterone or estradiol levels between those with and without depression, based on a prevalence of 5% and allowing for a 30% attrition rate. The total number analysed was 277 with that shortfall offset by a higher than expected number of participants exceeding the clinical cut-point for depression on the SMFQ (17% at year 3).(24) In order to describe changes in *urine* hormones over time, the area under the curve (AUC) with respect to the annual increase was calculated for each twelve-month time period using the trapezoid formula [21]. AUC calculation allows integration of urine hormone measures to bring four measures of urine hormones into one meaningful annualised measure. Two formulae were used to generate separate AUC data: AUC relative to ground (AUC_G_) and AUC relative to the increase (AUC_I_). AUC_G_ reflects *total* hormone output from baseline and thus the overall intensity of change. It is calculated using the sum of trapezoids calculated by the area under the curve formed by hormone levels at each time point. AUC_I_ which indicative of the change or *variability* in hormone levels over time as it ignores the distance from zero (as included in the AUC_G_). It is calculated by subtracting the area between zero and the first measure for the year of collection from the AUC_G_ [21]. As the AUC_I_ measures variability, and allows for negative values which would indicate a decrease in hormone concentrations [15].

Mixed-model linear regression analysis was used to account for repeated measurements to estimate a longitudinal relationship between the outcomes and exposure. Separate analyses were conducted for males and females, with testosterone and estradiol in both sexes. Mixed-models linear regression was also used to assess the relationship between the gender, time, and the outcome variables. Random intercepts for each subject were used in all mixed models to account for repeated measures in each adolescent. Analyses were conducted using Stata 16.1 (StataCorp, College Station, USA). With three primary outcomes (SMFQ score, YSR anxious/depressed, and YSR withdrawn/depressed subscales) that were interrelated, a modified Bonferroni correction was applied to the pre-specified significance of p = 0.05, halfway between no and full adjustment and set at p = 0.033.

### Ethics and consent

The ARCHER study has ethics approval from the Human Research Ethics Committee, University of Sydney (HREC 2010/13094, 2012/2425 and 2015/199) within the framework of the National Health and Medical Research Council Guidelines for Human Experimentation and consistent with the Declaration of Helsinki. A parent/guardian provided full written informed consent and adolescents provided assent prior to commencement in the study. The authors involved in data analysis did not have access to information that would directly identify study participants.

## Results

### Cohort characteristics

Of 342 adolescents recruited to the ARCHER study, 277 adolescents (158 males, 119 females) with complete or near-complete urine data were included in the mixed-models regression analyses. The mean (standard deviation (SD)) age at baseline was 11.8 (1.02) years for males and 11.6 (0.96) years for females (p = 0.23). The intended duration of follow-up for each participant in this study was three years and at the end 86% of females had reached menarche (2). There is no comparable measure in males however 134 of 158 (85%) males reached Tanner stage 4 or 5 at the end of the study.

The cohort demographics were similar to national data for age and sex, except for a higher percentage of Indigenous adolescents (11% v. 6%), and a slightly higher level of maternal education. The parent questionnaire included questions on chronic conditions, with only asthma (inhaler therapy only) and ADHD (treated and untreated) recorded. One endocrinologist (KS) reviewed all final growth curves. Height growth patterns (and self-reported Tanner stages) consistent with earlier, average, and later normal puberty were present. None were consistent with an endocrine abnormality.

### Longitudinal change in urine testosterone and estradiol hormone measures

All testosterone and estradiol results are centred on Tanner stage rather than against chronological age, given the wide variation in in Tanner stage for any given chronological age during puberty. The urine values are all higher than would be found in serum samples.

**Fig 1:**
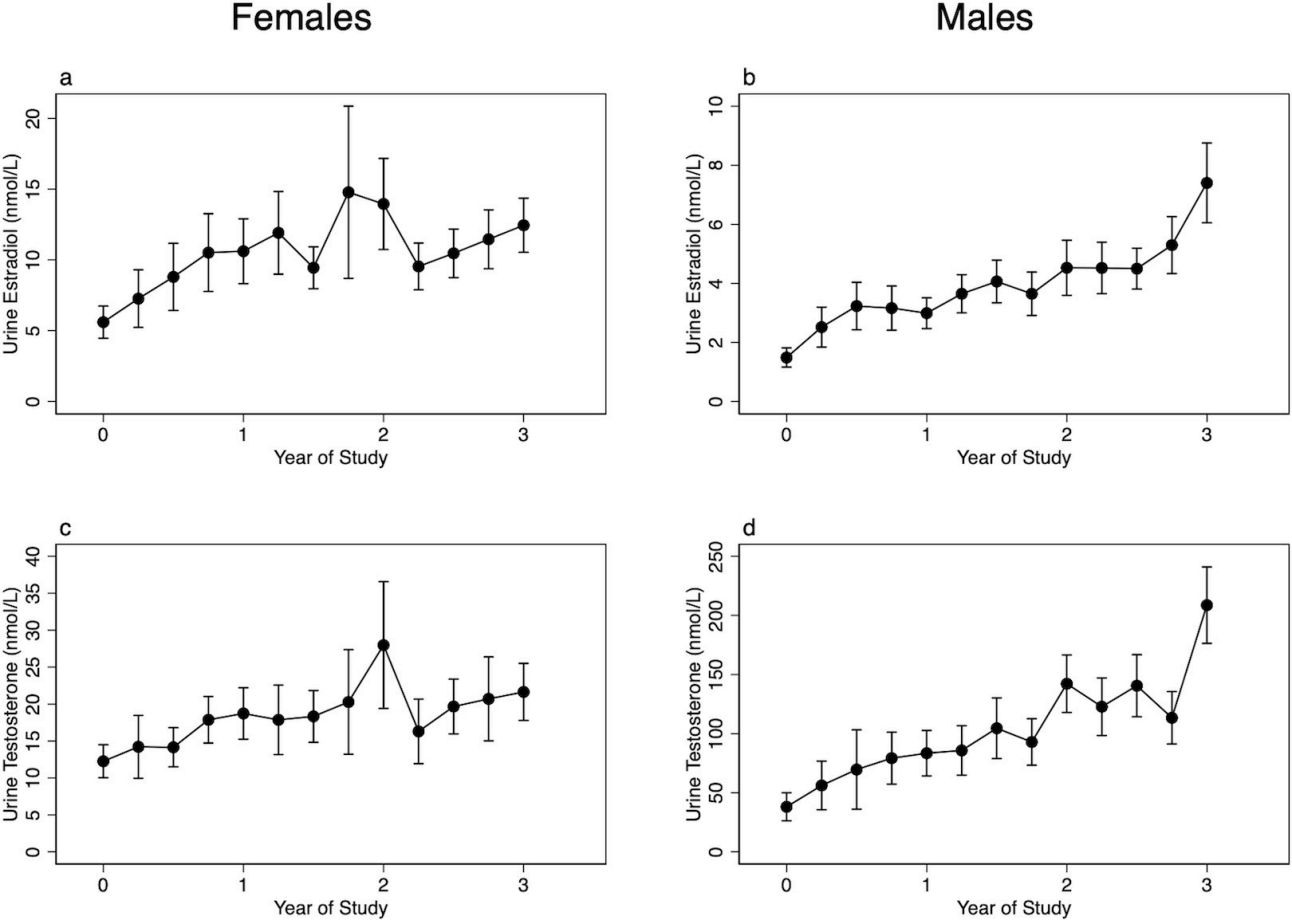
Mean three-month urine hormone levels for males and females. Urine hormone levels are corrected for specific gravity. * Note different y-axes for each sex/hormone combination. Urine hormones are measured via LC-MS/MS and reported in nmol/L. Error bars indicate 95% confidence intervals.

Fig 1 displays the mean urine hormone concentrations for each three-month collection. There were significant increases in both urine testosterone and estradiol over the collection period in both males and females (p<0.001 for all). Urine testosterone and estradiol were different between sexes across the course of the study (p<0.001 for all). Urine testosterone and estradiol were positively correlated for males (r = 0.53, p<0.001), and in females (r = 0.70, p<0.001).

Annual AUC_G_ (total) and AUC_I_ (variability) are provided in Table 1 and demonstrate some sex differences. There was a significant increase, as expected, in the AUC_G_ for testosterone and estradiol in both males and females (p<0.001 for all). In males, the AUC_G_ for testosterone and estradiol were positively correlated (r = 0.64, p<0.001), but not in females (r = 0.04, p = 0.52).

**Table 1:** Mean (SD) annual AUC_G_ and AUC_I_ for T and E_2_.

	Females (n = 119)			Time	Males (n = 158)			Time
	Year 1	Year 2	Year 3	p-value	Year 1	Year 2	Year 3	p-value
Testosterone								
AUC_G_ (nmol/L∙year)	14.8 (12.81)	21.5 (21.13)	21.6 (21.52)	<0.001	57.4 (99.34)	102.4 (118.05)	147.7 (135.84)	<0.001
AUC_I_ (nmol/L∙year)	3.0 (9.09)	3.3 (16.88)	-5.2 (25.46)	<0.001	22.2 (53.58)	21.1 (74.75)	10.0 (87.70)	0.15
Estradiol								
AUC_G_ (pmol/L∙year)	8.2 (6.48)	11.8 (10.41)	11.8 (8.88)	<0.001	2.4 (2.91)	3.8 (3.40)	5.3 (4.58)	<0.001
AUC_I_ (pmol/L∙year)	2.9 (7.48)	1.6 (15.14)	-3.4 (18.97)	<0.001	1.0 (1.95)	0.9 (2.66)	-3.3 (6.72)	<0.001

There was a significant decrease in variability—AUC_I_—for estradiol for both females and males (p<0.001 for both) over time. There was also a significant decrease in AUC_I_ for testosterone in females (p<0.001) but no significant change in males (p = 0.15). In both males and females AUC_I_ for testosterone and estradiol were both positively correlated (M: r = 0.33, p<0.001; F: r = 0.50, p<0.001).

### Longitudinal change in mood, behavior and sleep measures

The median values (interquartile range) for SMFQ, YSR and sleep variables are shown in Table 2. Except for sleep, higher values are less favourable. With respect to mood there were significant changes in the SMFQ and YSR anxious/depressed over the study, with values improving for males and deteriorating for females. The YSR withdrawn/depressed subscale followed a similar trend, but the sex divergence was not statistically significant. Females had significantly higher SMFQ scores compared to males across the study duration.

**Table 2:** Median values (and interquartile range) for mood and behaviour survey results.

	Females (n = 119)				Time	Males (n = 158)				Time	Sex
	Baseline	Year 1	Year 2	Year 3	p-value*	Baseline	Year 1	Year 2	Year 3	p-value*	p-value*
SMFQ	4 (2–8)	4 (2–7)	5 (2–9)	4 (2–13)	**<0.001**	3 (1–7)	2.5 (1–6)	2 (0.5–5)	2 (0–6)	**0.02**	**<0.001**
YSR total psychopathology	58 (28–84)	54 (24–76)	62 (28–84)	65 (31–89)	**0.03**	60 (34–87)	46 (24–81)	50 (21–81)	46 (21–79)	**<0.001**	0.30
YSR anxious/depressed	58 (50–81)	54 (50–81)	65 (50–89)	65 (50–93)	**<0.001**	58 (50–84)	54 (50–76)	54 (50–65)	54 (50–76)	**0.004**	0.12
YSR withdrawn/depressed	58 (50–79)	58 (50–69)	58 (50–79)	58 (54–87)	**0.001**	59.5 (50–76)	54 (50–65)	54 (50–76)	54 (50–65)	**0.003**	0.04
YSR somatic	76 (58–90)	65 (54–90)	76 (54–90)	65 (54–90)	0.07	79 (54–92)	58 (54–87)	58 (50–87)	58 (54–79)	**<0.001**	0.07
YSR social	69 (50–87)	58 (54–79)	69 (50–87)	58 (54–87)	0.64	65 (50–87)	54 (50–81)	54 (50–81)	54 (50–76)	**0.004**	0.05
YSR thought	79 (58–90)	73 (54–90)	73 (58–90)	73 (58–92)	0.40	79 (62–96)	69 (54–89)	62 (50–89)	62 (50–84)	**<0.001**	0.36
YSR attention	62 (50–76)	62 (50–76)	62 (50–76)	62 (54–84)	0.11	58 (50–84)	56 (50–76)	58 (50–76)	54 (50–76)	0.19	0.21
YSR rule break	54 (50–65)	54 (50–65)	54 (50–65)	58 (50–76)	**<0.001**	54 (50–73)	54 (50–73)	54 (50–73)	54 (50–79)	**0.001**	0.04
YSR aggressive	54 (50–69)	50 (50–62)	54 (50–62)	54 (50–69)	0.32	54 (50–73)	50 (50–65)	50 (50–65)	50 (50–73)	0.26	0.57
Impulsive-aggression	3 (1–4)	2 (1–4)	2 (1–4)	3 (1–4)	0.99	3 (1–5)	3 (1–4)	2 (1–5)	2 (1–4)	0.05	0.87
Sleep–weekday (hr)	10 (9–10.75)	9.5 (8.5–10)	9 (8–10)	8.625 (8–9.5)	**<0.001**	9.5 (8.5–10.5)	9 (8.5–10)	9 (8–10)	8.85 (8–9.5)	**<0.001**	0.42
Sleep—weekend (hr)	9.5 (8.5–10.5)	9.5 (8–10)	9 (8–10)	9 (8–10)	**0.001**	9 (8–10.5)	9 (8–10)	8.5 (7–10)	9 (7.5–10)	0.14	**0.01**

*Time p-value is from a mixed regression model assessing the relationship between year and the mood and behaviour survey results, respectively by sex.

**Sex p-value is from a linear mixed regression model assessing the relationship between sex, year and the mood and behaviour survey results.

SMFQ = Short Mood and Feelings Questionnaire; YSR = Youth Self Report.

With respect to other behaviors the YSR total pathology scale improved in males and deteriorated in females. Males had significant changes in their somatic complaints, social problems, thought problems, and rule-breaking behavior subscales, with median scores improving across the study duration. Females had significant deterioration in their rule-breaking behavior subscale only. Both sexes had significant decreases in the amount of self-reported sleep during weekdays over the study. Females progressively slept less on weekend nights. These symptom scores are mostly subthreshold for clinical diagnosis, and reflect the multiple epidemiological studies that show an upswing of psychopathology in females but not in males during adolescence [16–18], and indicate that our sample has the trends observed in the wider community.

### Relationships between mood and behavior measures and urine hormones

#### Total testosterone and estradiol changes

In males, a significant positive association was observed for urine testosterone and estradiol AUC_G_ with the YSR rule-breaking behavior subscale. Negative associations were observed for both urine testosterone and estradiol AUC_G_ and sleep on weekdays.

In females, no significant associations were observed for AUC_G_ for urine estradiol and any mood or behavioral outcome. Females had significant positive associations between total change in urine testosterone (as measured by AUC_G_) and YSR anxious/depressed and withdrawn/depressed subscales. Significant negative associations were observed for AUC_G_ for testosterone and self-reported weekday and weekend sleep for females. These relationships are shown in Table 3.

**Table 3:** Relationships between survey outcomes and hormone Area Under the Curve Relative to Ground (AUC_G_).

	Females				Males				
	AUC_G_ for E_2_		AUC_G_ for T			AUC_G_ for E_2_		AUC_G_ for T	
	Beta	p-value*	Beta	p-value*		Beta	p-value*	Beta	p-value*
SMFQ	0.023	0.51	0.034	0.05		0.051	0.30	-0.0003	0.85
YSR total psychopathology	0.158	0.31	0.164	0.06		0.217	0.49	-0.001	0.89
YSR anxious/depressed	0.132	0.20	**0.139**	**0.01**		-0.208	0.28	-0.008	0.19
YSR withdrawn/depressed	0.057	0.57	**0.172**	**0.001**		0.095	0.61	-0.006	0.35
YSR somatic complaints	0.048	0.63	0.048	0.38		-0.411	0.05	-0.010	0.05
YSR social problems	0.055	0.58	0.025	0.65		0.023	0.90	-0.003	0.59
YSR thought problems	0.035	0.72	0.109	0.05		-0.247	0.21	-0.009	0.15
YSR attention problems	0.054	0.55	0.056	0.24		-0.010	0.96	-0.003	0.67
YSR rule-breaking behaviours	0.117	0.13	0.075	0.06		**0.594**	**0.001**	**0.012**	**0.03**
YSR aggressive behaviours	0.113	0.14	0.076	0.09		0.276	0.12	-0.002	0.71
YSR self-harm	0.001	0.52	0.001	0.55		-0.001	0.64	-0.0001	0.17
YSR suicidal ideation	0.002	0.44	0.001	0.19		0.005	0.20	-0.0001	0.67
Impulsive-aggression	0.006	0.70	0.006	0.50		0.023	0.46	-0.0005	0.61
Sleep–weekday (hr)	-0.003	0.70	**-0.017**	**<0.001**		**-0.069**	**<0.001**	**-0.002**	**<0.001**
Sleep–weekend (hr)	-0.017	0.12	**-0.015**	**0.01**		0.0001	0.99	0.0001	0.94
Self-harm	0.001	0.07	0.0004	0.10		-0.0001	0.93	-0.00001	0.61

*p-value is from a linear mixed regression model assessing the relationship between survey outcomes and hormone AUC_G_, separately by sex and hormone.

SMFQ = Short Mood and Feelings Questionnaire; YSR = Youth Self Report.

#### Annual hormone variability in testosterone and estradiol

In females there were significant inverse relationships between urine hormone variability (measured by AUC_I_) and mood and behavioral problems. Greater variability of both urine estradiol and testosterone was associated with lower YSR total psychopathology, anxious/depressed, and social problems scores. Greater variability in urine estradiol only was associated with both lower YSR attention problems and impulsive-aggressive behaviors in females. Testosterone variability in females was negatively associated with self-harm behaviors. In males, the only significant finding in hormone variability was a negative association between estradiol and sleep at weekends. These relationships are shown in Table 4.

**Table 4:** Relationships between survey outcomes and annual hormone Area Under the Curve Relative to Increase (AUC_I_).

	Females					Males					
	AUC_I_ for E_2_		AUC_I_ for T			AUC_I_ for E_2_			AUC_I_ for T		
	Beta	p-value*		Beta	p-value*		Beta	p-value*		Beta	p-value*	
SMFQ	-0.029	0.17		-0.015	0.28		0.025	0.45		0.003	0.17	
YSR total psychopathology	**-0.293**	**0.001**		**-0.156**	**0.01**		0.178	0.35		-0.005	0.69	
YSR anxious/depressed	**-0.182**	**0.002**		**-0.088**	**0.03**		0.051	0.69		-0.003	0.74	
YSR withdrawn/depressed	-0.124	0.04		-0.061	0.13		0.138	0.27		-0.001	0.89	
YSR somatic complaints	0.095	0.11		-0.073	0.06		0.126	0.36		0.004	0.72	
YSR social problems	**-0.132**	**0.03**		**-0.084**	**0.03**		0.084	0.46		0.009	0.24	
YSR thought problems	-0.100	0.09		**-0.085**	**0.03**		0.040	0.75		-0.004	0.67	
YSR attention problems	**-0.119**	**0.03**		-0.066	0.06		0.028	0.81		-0.012	0.14	
YSR rule-breaking behaviours	**-0.096**	**0.04**		-0.053	0.09		-0.030	0.79		-0.005	0.57	
YSR aggressive behaviours	**-0.111**	**0.02**		-0.035	0.24		-0.023	0.84		-0.008	0.30	
YSR self-harm	-0.001	0.28		-0.001	0.10		-0.001	0.77		-0.0001	0.53	
YSR suicidal ideation	-0.003	0.07		-0.001	0.20		0.002	0.48		-0.0001	0.73	
Impulsive-aggression	**-0.021**	**0.02**		-0.007	0.24		0.015	0.43		-0.0002	0.89	
Sleep–weekday (hr)	0.008	0.11		0.003	0.40		-0.008	0.50		-0.001	0.34	
Sleep—weekend (hr)	0.007	0.25		0.007	0.12		**-0.047**	**0.01**		-0.001	0.43	
Self-harm	-0.001	0.27		**-0.001**	**<0.001**		-0.0002	0.72		0.00002	0.70	

*p-value is from a linear mixed regression model assessing the relationship between survey outcomes and hormone AUC_I_, separately by sex and hormone. Boldface indicates a significant (p<0.033) relationship.

## Discussion

This study suggests that the associations of these sex hormones, iso-sex and cross-sex hormones, with adolescent mood and behaviour are more complex when multiple outcomes and patterns of change are considered. Some significant associations described are not always in agreement with previous studies on this topic. This may be due to the cross-sectional nature of many previous studies assessing only a single time point during puberty as well as less accurate laboratory measures for sex steroids, and without consideration of cross-sex hormones.

Of clinical importance is the association of higher urinary testosterone and lower estradiol variability in females with anxiety, depression, and withdrawn mood. In support of our finding is a re-analysis of data from the Great Smoky Mountain Study (GMS), a longitudinal psychiatric epidemiology study. Estradiol levels from dried blood spots, based on random sampling for time of day or menstrual cycle, and less frequently than in the present study, were positively associated with depression in females. A subsequent re-analysis of these dried blood spots for testosterone found a stronger relationship for testosterone with depression in females [22]. Our study demonstrated less variability in estradiol levels and higher absolute testosterone levels have negative associations with affect. This combination is frequently observed together with affect disturbance in hyperandrogenic states in females such as insulin resistance and the polycystic ovary syndrome [23,24]. Thus, these conditions may benefit by reduction in testosterone exposure and a more cyclical estradiol production, either by pharmacotherapy or by restoration of ovarian cyclicity with lifestyle intervention and weight reduction. Clinical trial data are needed to support this proposition.

Our study was unable to demonstrate associations of testosterone with aggressive behaviors with the normal increases of this hormone during puberty. The two significant associations with total testosterone in males were a positive one with YSR- rule breaking scale and a negative one with sleep duration. Goddings et al recently reported on a cross-sectional study measuring salivary testosterone, self-reported tendencies on risk taking in males and fMRI neural activation that provides support our longitudinal observations if risk taking is viewed as one aspect of rule breaking. It is conceivable that our data may be useful in the interpretation of the numerous studies now considering the impact of puberty on neurocognition [10–12,25].

The study data may also be useful clinically for in three other ways. First, results provide reassurance for parents that induction of puberty in hypogonadal males should not increase aggressive behaviours [26,27]. In males the absolute increase in urine testosterone was associated with increased rule breaking, but not with measures of aggression or mood disturbance. This finding was a conclusion hinted at in a previous systematic review [1]. It is likely that the adverse effects of testosterone, which were not apparent in community adolescents in this study, have been extrapolated from androgen abuse in adult males with often massive supra-physiological dosing and where anger, anxiety and low mood are features [28,29]. Secondly, cross-sex hormone data combined with same sex hormone data may also be useful for understanding the effects of gender affirming hormone therapy in adolescents with gender dysphoria [30]. Thirdly, these data may direct clinicians and parents to consider alternative explanations for concerning adolescent behaviours. The hormones remain at the same level after puberty, but behaviours generally ameliorate, an observation that may fit better with neurocognitive maturation extending into the early 20s. As an extension, alternative management approaches should be considered. These might include interventions around improving self-regulation for emotions. Alternatively, exogenous causes for anger and /or aggression because of depression or distress caused by others may need to be addressed. This would include assessment of less than ideal sleep patterns that in both sexes show some negative associations with puberty hormones and may amplify the classic sleep architecture changes during adolescence [31].

The strengths of this study are its advantages over other recent adolescent cohort studies [3,4,32] with an intensive biological sampling protocol with precise timing of sample collection and hormone measurements with the current gold standard in order to describe the progression of puberty. That we achieved superior, near complete longitudinal biological data collection using urine specimens [14] highlights the need for ‘adolescent friendly’ research protocols to answer important research questions in this age group. The limitations were first the relatively short period of observation, three years for an individual meant not all participants reached pubertal maturity and some were already well advanced in puberty at recruitment. The lower age limit for recruitment was to ensure the participants were able to provide their own mood and behavior assessments. It is also acknowledged that there are external influences on mental health and wellbeing, but much larger numbers would have been required and the resources, especially for hormone measures, beyond the limit of most granting bodies.

## Conclusion

This study provides a more nuanced view of the progression of puberty hormone change and its impacts on mood and behaviour during puberty. Our original hypothesis was part proven and variability in change had a minor role, not rate of change per se. Without any implications for any hormonal interventions during normal puberty, the present study provides clinically relevant data that supporting an interpretation of impact of hyperandrogenemia in female affective disorders., There are possible implications for the use of gonadal hormone replacement in hypogonadism and in affirmative management of gender dysphoria but this would require specific studies designed to test any hypotheses. There is a final important health message in our findings. Puberty is only one part of the complex developmental stage of adolescence that includes psychosocial transitions and neurocognitive maturation. If mood and behavioural changes are attributed solely to hormones in the second decade many young people will be deprived of health care interventions that would go far towards improving their health and wellbeing.
